# Haralick texture features from apparent diffusion coefficient (ADC) MRI images depend on imaging and pre-processing parameters

**DOI:** 10.1038/s41598-017-04151-4

**Published:** 2017-06-22

**Authors:** Patrik Brynolfsson, David Nilsson, Turid Torheim, Thomas Asklund, Camilla Thellenberg Karlsson, Johan Trygg, Tufve Nyholm, Anders Garpebring

**Affiliations:** 1 Dept. of Radiation Sciences, Umeå University, Umeå, Sweden; 2 Computational Life Science Cluster (CLiC), Department of Chemistry, Umeå University, Umeå, Sweden; 3 Cancer Research UK Cambridge Institute, University of Cambridge, Cambridge, United Kingdom

## Abstract

In recent years, texture analysis of medical images has become increasingly popular in studies investigating diagnosis, classification and treatment response assessment of cancerous disease. Despite numerous applications in oncology and medical imaging in general, there is no consensus regarding texture analysis workflow, or reporting of parameter settings crucial for replication of results. The aim of this study was to assess how sensitive Haralick texture features of apparent diffusion coefficient (ADC) MR images are to changes in five parameters related to image acquisition and pre-processing: noise, resolution, how the ADC map is constructed, the choice of quantization method, and the number of gray levels in the quantized image. We found that noise, resolution, choice of quantization method and the number of gray levels in the quantized images had a significant influence on most texture features, and that the effect size varied between different features. Different methods for constructing the ADC maps did not have an impact on any texture feature. Based on our results, we recommend using images with similar resolutions and noise levels, using one quantization method, and the same number of gray levels in all quantized images, to make meaningful comparisons of texture feature results between different subjects.

## Introduction

Texture analysis was developed in the 1970s as a method for image analysis and classification^1^. It is a way of describing the spatial distribution of intensities^2^, which makes it useful in classification of similar regions in different images. In medical image analysis, texture analysis was adopted for analysis of ultrasound images of the liver^3^ and heart^4^ in the late 1970s and early 1980s, and gained popularity in the 1990s and 2000s for many medical imaging application, including oncology. Texture analysis enables description of tissue heterogeneity, a property believed to influence the outcome of cancer treatment^5^, which has led to applications in treatment response evaluation^6, 7, 5, 8^. Haralick texture features^1, 9, 10^ calculated from a gray level co-occurrence matrix (GLCM) is a common method to represent image texture, as it is simple to implement and results in a set of interpretable texture descriptors^1, 11^ Although a large and increasing number of studies uses Haralick’s features to analyze texture in magnetic resonance images (MRI) and images from other modalities^9, 12–15^ there is no standardized way of performing these analyzes^13^. For example, GLCM texture analysis requires that the images must be quantized to a given number of gray levels^1^. Different groups tend to use apparently arbitrarily chosen quantization methods when constructing the GLCM, although the gray level quantization could affect the calculated texture features^15–17^.

Leijenaar *et al*.^13^ examined the effect of windowing method, i.e. how the images were quantized into gray level bins, on GLCM features derived from standardized uptake value (SUV) maps from positron emission tomography (PET) images. They compared results using a fixed width of the gray level bins to results using a fixed number of bins, and concluded that a fixed width was preferred when comparing texture feature values between subjects. Torheim *et al*.^8^ used four Haralick features to predict treatment outcome for 81 cervical cancer patients using pharmacokinetic parameter maps based on dynamic contrast enhanced (DCE) MRI. This study did not specifically focus on the effect of GLCM construction parameters, but tested seven different numbers of bins (4, 8, 16, 32, 64, 128 and 256) as part of the experimental design. They found that the quantization used for constructing the GLCM had a significant impact on the accuracy of the prediction models. Gómez *et al*.^15^ investigated the effect of number of gray level bins as well as other GLCM settings on the discriminating power of 22 texture features in breast ultrasound images. They use a fixed gray level range for all images, regardless of the range in each individual image. This study did not show how individual features varied, but found that gray level quantization did not significantly affect the discrimination power of the texture features. A few studies tested the effect of MR imaging parameters on texture features. Savio *et al*.^18^ found that slice thickness did not significantly alter GLCM features based on MR scans of multiple sclerosis patients. Mayerhoefer *et al*.^19^ did a phantom study to assess the effect of MR acquisition parameters: number of scan averages, repetition time (TR), echo time (TE) and receiver bandwidth on a variety of texture features, including 11 GLCM based features. They found that GLCM features were generally sensitive to variation in these parameters, and that this sensitivity increased with spatial resolution. However, the GLCM outperformed the other features in discriminating between physiological patterns in the images. This indicated that even though the features vary, they maintain the ability to discriminate between relevant image patterns. The same group also investigated the effect of MRI interpolation, where they used three different methods to increase the spatial resolution in their phantom images^20^. Materka and Strzelecki^21^ studied how inhomogeneity in MR images, caused by e.g. magnetic field bias, affect texture features. They found that texture features could be sensitive to inhomogeneity, as the variation in intensities could obscure the underlying texture. They recommend correcting for inhomogeneity before texture analysis. However, they remarked that some GLCM features were more robust than others to non-uniformities.

In the last couple of years, several studies using texture analysis based on gray level co-occurrence matrices of diffusion weighted (DW) MRI have been published. These include studies of gliomas^7, 22^ prostate cancer^23, 24^ renal cell carcinomas^25^ and breast cancer^26^. The most common approach in these studies was to analyze textures in the apparent diffusion coefficient (ADC) maps. These maps were constructed based on DW images acquired using several b-values^27^. The range and number of b-values used to construct the ADC map will affect the resulting ADC values, due to intravoxel incoherent motion^28, 29^ and the accuracy of the fit to the data. To our knowledge, there are no publications describing how the choice of b-values for ADC calculation affect GLCM texture features.

Despite the large interest in applications of texture analysis to aid detection, diagnosis and treatment response assessments, there is no consensus or standards regarding the texture analysis workflow of medical images in general, or in the reporting of crucial parameters such as gray level quantization method or the number of gray level used to create the GLCM.

We have two aims with this study. Firstly, we wanted to assess how sensitive Haralick texture features are to the choice of imaging parameters, such as noise level, resolution and ADC map construction, and to methods of gray level quantization used to generate the GLCM and the Haralick texture features. When planning e.g. multi-center studies or when replicating or implementing a published method it is important to know which texture features are sensitive to variations in imaging settings or analysis methods. Secondly, we wanted to compile a set of recommendations for how to preform texture analysis on ADC maps. To investigate if the results are dependent on different tumor types and anatomical regions we investigated the texture feature variations on a data set comprising 72 delineated high-grade gliomas and a data set comprising 36 delineated tumors in patients diagnosed with high risk prostate cancer.

## Results

**Figure 1:**
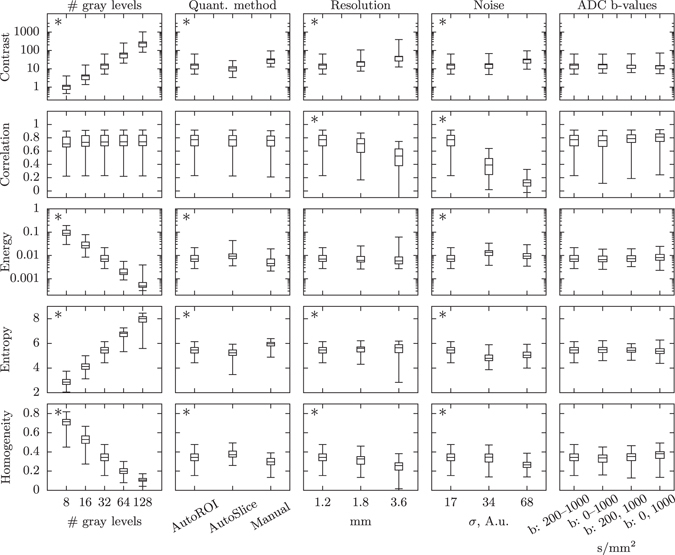
Changes in texture feature distributions with different imaging and pre-processing settings. The box plots show the distribution of contrast, correlation, energy, entropy and homogeneity for the 72 ROIs in the glioma data set. The box shows the first and third quartiles, with the median value indicated by the center line. The whiskers show the extreme values. An asterisk in the upper left corner indicates that at least one pair of settings is significantly different.

We varied five imaging and pre-processing parameters; noise level, resolution, how the ADC map was constructed, quantization method, and the number of gray levels in the quantized images; to observe how they influence the resulting Haralick texture features. Figure 1 shows box plots of five commonly used Haralick features; contrast, correlation, energy, entropy and homogeneity, for 72 regions of interest (ROIs) of the glioma data set. Contrast, entropy and homogeneity changes significantly with all varied parameters except the b-values related to ADC map construction. Energy is significantly affected by changes in the number of gray levels, quantization levels and noise, whereas correlation changes significantly only with resolution and image noise.

**Figure 2:**
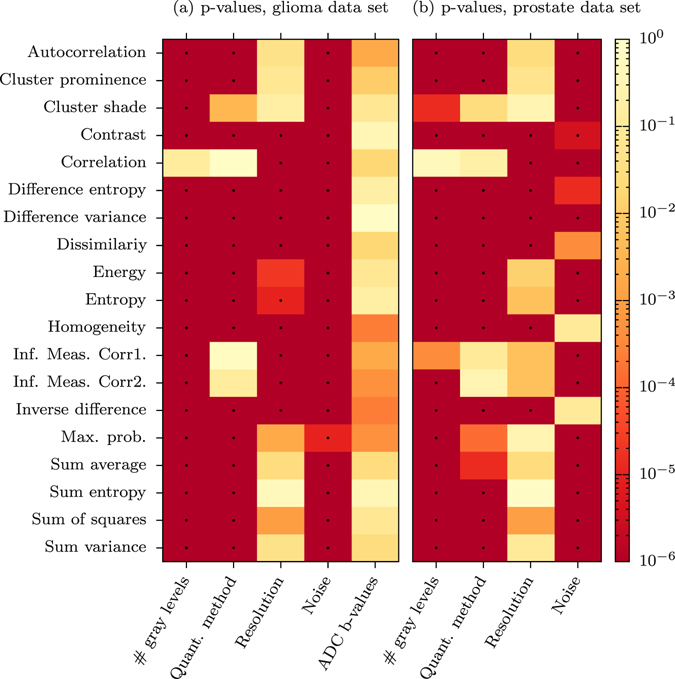
Probabilities that texture features are unaffected by changes in imaging or pre-processing steps. Heatmaps showing the probability (p-value) that all settings for a given parameter give the same texture feature values. The dots represent significant changes at the *α* = 0.01 level, with Bonferroni corrections. (**a**) Shows the result from the glioma data set, (**b**) from the prostate cancer data set.

Figure 2 is a heatmap showing the probabilities that all settings for a given parameter give the same texture feature values. Figure 2(a) shows the results from the glioma data set, and Fig. 2(b) shows the result from the prostate cancer datatset. The two leftmost columns in each heatmap show pre-processing steps when generating the GLCM, and column three to five in (a) and three to four in (b) show imaging settings. GLCM size has the largest effect on all texture features except for correlation in both data sets, and cluster shade and information measure of correlation 1 in the prostate data set. The quantization method has a significant effect on most features in both data sets. Resolution significantly affect the values for about half of the features. Noise has a significant effect on all features in the glioma data set, and most features in the prostate data set. The choice of b-values used for constructing the ADC maps in the glioma data set had no significant effect on any feature.

## Discussion

We set out to find how sensitive Haralick texture features of ADC maps are to the choice of imaging parameters and texture analysis parameters. Our main findings were that the choice of GLCM size, DWI noise, resolution and quantization method significantly affect the values of the resulting texture features. The combination of b-values used to construct the ADC map had no significant impact on any texture feature. The results were very similar for ADC maps of both glioma and prostate tumors.

GLCM size has overall the largest impact on the texture features values, exemplified in Fig. 1, where contrast and energy changes by several orders of magnitude, and entropy and homogeneity changes by approximately a factor of 10. This is not surprising, considering the properties of the GLCM and how the texture features are calculated. Each texture feature is a function of the normalized GLCM, *p*(*i*, *j*), where the values are mostly determined by the row and column indices (*i*, *j*) of the GLCM, see Table 3. A large-size GLCM will have larger values of (*i*, *j*) and thus a big impact on the values of most textures. Further, the elements of a normalized GLCM will sum to one: ${\sum }_{i,j}p(i,j)=1$, which means that as the GLCM gets larger, each element value *p*(*i*, *j*) will get smaller. For example, homogeneity, which is weighted by the inverse of the indices and linearly with *p*(*i*, *j*) will have both of these effects working in the same direction, making it heavily dependent on GLCM size, which can be seen in Fig. 1. One way of decreasing the influence of the GLCM size on the values of the texture features is to normalize the indices, $(i,j)\to (i/N,j/N)$ where *N* is the number of gray levels. This has previously been used by Soh *et al*.^17^ to compare texture features across different quantization schemes, and Clausi *et al*.^16^ introduced two normalized features to improve classification.

**Figure 3:**
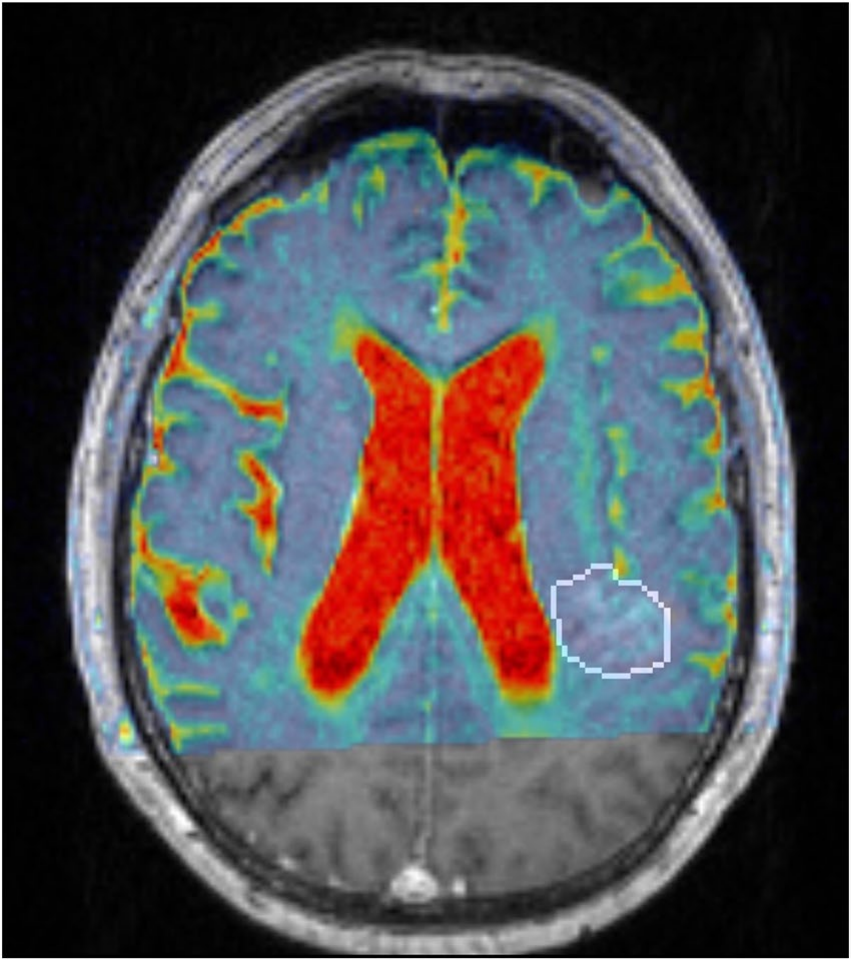
The effect of ROI uncertainties to the texture features. The delineated glioma in a slice near the left lateral ventricle in a 73 year old male, from which the variations in the texture features were calculated in Table 1. The colormap shows the ADC map, fused on the T1-weighted contrast enhanced MPRAGE. An expansion or a shift by one voxel can include CSF in the ROI, which will increase the minimum and maximum values in the ROI, and will have an effect on the resulting texture features. The manual quantization method is less sensitive to this shift.

The quantization method had a significant impact on 15 of 19 features is the glioma data set and 13 of the 19 features in the prostate data set. The choice of how to quantize the images impacts the values of the texture features and different methods should be considered depending on what the underlying images show. Figure 6 shows a large spread in the range of ADC values in the ROIs for both the glioma and prostate data set. A value above 3000 mm^2^/s in an ROI usually indicates liquid, such as cerebro-spinal fluid (CSF) in the brain or urine in the prostate region, and a value of 0 mm^2^/s indicates a region with no signal. In patients where the ROI is close to the ventricles or the bladder, the texture feature values can be very dependent on the definition of the ROI if the AutoROI or AutoSlice methods are used. An example of this is shown in Table 1, where the change in texture features for AutoROI and manual quantization methods were calculated when expanding the ROI with one voxel in a slice from the glioma data set where the tumor is close to the left lateral ventricle, see Fig. 3. A change of one voxel can be due to e.g. inter-operator variability, or a registration error. The minimum and maximum values inside the ROI changed from 310–1344 mm^2^/s to 209–1827 mm^2^/s due to the expansion. Of the 19 features, only cluster prominence and maximum probability showed a smaller change in feature values using AutoROI. Hence, if the ROI is close to values much larger or smaller than inside the ROI, a manual quantization method should be used.

**Table 1:** Percentage change in texture features when expanding the ROI by one voxel.

	AutoROI (%)	Manual (%)	Manual/AutoROI
Autocorrelation	−40.8	4.59	0.112
Cluster Prominence	−53.9	111	2.05
Cluster shade	−8840	286	−0.0323
Contrast	−43.9	26.1	−0.595
Correlation	−5.91	−5.31	0.898
Difference entropy	−9.69	3.45	−0.356
Difference variance	−38.5	35.8	−0.930
Dissimilarity	−0.276	0.0886	−0.321
Energy	57.8	30.3	0.525
Entropy	−6.32	1.50	−0.237
Homogeneity	38.8	3.53	0.0910
Information measure of correlation 1	−25.8	−11.3	0.437
Information measure of correlation 2	−10.1	−2.95	0.293
Inverse difference	25.1	1.13	0.0449
Maximum probability	0.422	25.5	60.5
Sum average, *μ*_*x*+*y*_	−22.5	−4.19	0.186
Sum entropy	−8.18	0.386	−0.0472
Sum of squares	−48.2	17.6	−0.366
Sum variance	−49.3	15.4	−0.312

The sensitivity of AutoROI and a manual level of 500 and 1500 mm^2^/s to the definition of the ROI for a patient with a tumor close to the left lateral ventricle, in the slice shown in Fig. 3.

If CSF and signal void are believed to be important features in the ROI of brain tumors, a limit like 0 – 3000 mm^2^/s might be used. A narrower ADC range such as 500–1500 mm^2^/s contains white matter, gray matter and tumors but excludes CSF and signal voids. This approach minimizes the variations caused by uncertainties in the ROI, and decreases the likelihood of discretizing so that important texture features are hidden, as shown in Fig. 5.

Resolution had a significant impact on about half the features, see Fig. 2. As can be expected, the difference between 1.2 and 1.8 mm is not very large, whereas the difference between 1.2 and 3.6 mm is significant in all features affected by resolution, see Fig. 1.

Noise had a small but significant impact on all features for the glioma data set and a significant impact on 15 of 19 features of the prostate data set. The difference is most likely due to the inherent difference in the signal to noise ratio, SNR, in the tumors between the two data sets.

Mayerhoefer *et al*.^19^ assessed how the number of scan averages, repetition time, echo time and receiver bandwidth affected 11 Haralick features. They conclude that “*variations in MRI protocols lead to considerable differences in texture features*”. Number of scan averages, echo time and receiver bandwidth all affect the SNR, and our findings are in line with Mayerhoefer *et al*.’s results. Leijenaar *et al*.^13^ examined the effect of quantization methods on SUV maps from PET images. They suggest keeping the gray level step size fixed, i.e. the number of gray levels in the original image that will be assigned the same gray level in the quantized image. This is done by varying both the minimum and maximum values and the number of gray levels (GLCM size). Based on our results we recommend keeping both the minimum and maximum values and the GLCM size fixed. This will also keep the step size fixed, but will not introduce large variations of the texture features as a result of varying the GLCM size.

In summary, to meaningfully compare texture feature values of quantitative data such as ADC maps between patients, we have the following recommendations:

- **Use images with similar resolution and noise levels**.
- **Use one quantization method**. With quantitative data, such as ADC maps, a manual limit is preferable. Find the range of intensities that are common for all data sets in the cohort, or that reflect the anatomy or information of interest, and use that as a common limit for all images.
- **Use one GLCM size**. The number of gray levels should be chosen so that intensity variations in the relevant regions are resolved. A large range of ADC values should accompany a larger GLCM.
- **Report settings**. Report image resolution, image SNR, GLCM size and quantization method when publishing models using texture analysis.

## Theory

### Texture analysis

**Figure 4:**
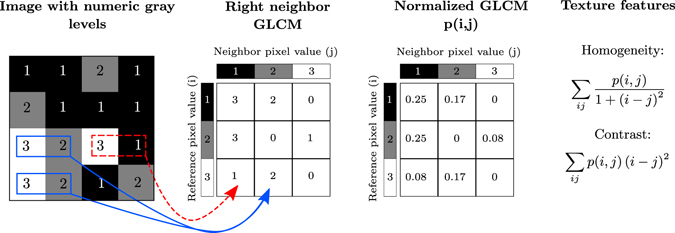
A description of how Haralick’s texture features are calculated. In an example 4 × 4 image ROI, three gray levels are represented by numerical values from 1 to 3. The GLCM is constructed by considering the relation of each voxel with its neighborhood. In this example we only look at the neighbor to the right. The GLCM acts like a counter for every combination of gray level pairs in the image. For each voxel, its value and the neighboring voxel value are counted in a specific GLCM element. The value of the reference voxel determines the column of the GLCM and the neighbor value determines the row. In this ROI, there are two instances when a reference voxel of 3 “co-occurs” with a neighbor voxel of 2, indicated in solid blue, and there is one instance of a reference voxel of 3 with a neighbor voxel of 1, indicated in dashed red. The normalized GLCM represents the frequency or probability of each combination to occur in the image. The Haralick texture features are functions of the normalized GLCM, where different aspects of the gray level distribution in the ROI are represented. For example, diagonal elements in the GLCM represent voxels pairs with equal gray levels. The texture feature “contrast” gives elements with similar gray level values a low weight but elements with dissimilar gray levels a high weight. It is common to add GLCMs from opposite neighbors (e.g. left-right or up-down) prior to normalization. This generates symmetric GLCMs, since each voxel has been the neighbor and the reference in both directions. The GLCMs and texture features then reflect the “horizontal” or “vertical” properties of the image. If all neighbors are considered when constructing the GLCM, the texture features are direction invariant.

Haralick texture features are calculated from a Gray Level Co-occurrence Matrix, (GLCM), a matrix that counts the co-occurrence of neighboring gray levels in the image. The GLCM is a square matrix that has the dimension of the number of gray levels *N* in the region of interest (ROI). Figure 4 gives an overview of how the GLCM is constructed and how the texture features are calculated.

Each texture feature is a function of the elements of the GLCM, and represents a specific relation between neighboring voxels. The texture features can indicate e.g. image contrast (large differences between neighboring voxels) or entropy, (the orderliness of the gray level distribution in the image). Tables 2 and 3 show how the texture features are defined.

**Table 2:**
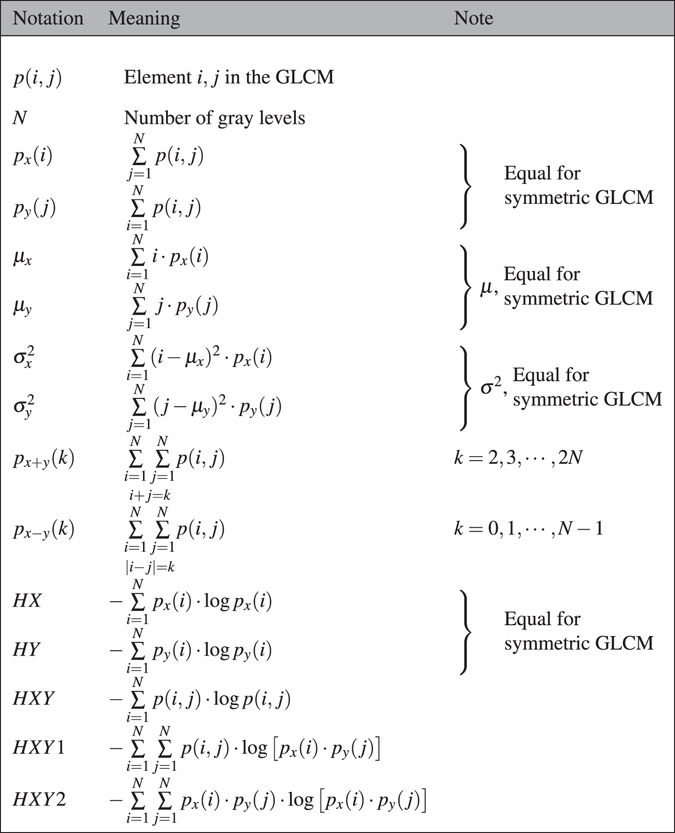
Variables and notation used to calculate Haralick features.

**Table 3:** Haralick texture features calculated from GLCMs. There was an error in the definition of *Sum variance* in Haralick *et al*.^1^, which has been corrected.

Feature	Equation	Ref.
Autocorrelation	$\sum _{i=1}^{N}\sum _{j=1}^{N}(i\cdot j)p(i,j)$	17
Cluster Prominence	$\sum _{i=1}^{N}\sum _{j=1}^{N}{(i+j-2\mu )}^{3}p(i,j)$	1
Cluster shade	$\sum _{i=1}^{N}\sum _{j=1}^{N}{(i+j-2\mu )}^{4}p(i,j)$	1
Contrast	$\sum _{i=1}^{N}\sum _{j=1}^{N}{(i-j)}^{2}p(i,j)$	1
Correlation	$\sum _{i=1}^{N}\sum _{j=1}^{N}\frac{(i\cdot j)p(i,j)-{\mu }_{x}{\mu }_{y}}{{\sigma }_{x}{\sigma }_{y}}$	1
Difference entropy	$-\sum _{k\mathrm{=0}}^{N-1}{p}_{x-y}(k)\mathrm{log}\,{p}_{x-y}(k)$	1
Difference variance	$\quad \sum _{k\mathrm{=0}}^{N-1}{(k-{\mu }_{x-y})}^{2}{p}_{x-y}(k)$	1
Dissimilarity	$\quad \sum _{i\mathrm{=1}}^{N}\sum _{j\mathrm{=1}}^{N}|i-j|\cdot p(i,j)$	17
Energy	$\quad \sum _{i\mathrm{=1}}^{N}\sum _{j\mathrm{=1}}^{N}p{(i,j)}^{2}$	1
Entropy	$-\sum _{i\mathrm{=1}}^{N}\sum _{j\mathrm{=1}}^{N}p(i,j)\mathrm{log}\,p(i,j)$	1
Homogeneity	$\quad \sum _{i\mathrm{=1}}^{N}\sum _{j\mathrm{=1}}^{N}\frac{p(i,j)}{1+{(i-j)}^{2}}$	17
Information measure of correlation 1	$\quad \frac{HXY-HXY1}{\max (HX,HY)}$	1
Information measure of correlation 2	$\quad \sqrt{1-\exp [-2(HXY2-HXY)]}$	1
Inverse difference	$\quad \sum _{i\mathrm{=1}}^{N}\sum _{j\mathrm{=1}}^{N}\frac{p(i,j)}{1+|i-j|}$	16
Maximum probability	$\mathop{\max }\limits_{i,j}p(i,j)$	17
Sum average, ${\mu }_{x+y}$	$\quad \sum _{k\mathrm{=2}}^{2N}k{p}_{x+y}(k)$	1
Sum entropy	$-\sum _{k\mathrm{=2}}^{2N}{p}_{x+y}(k)\mathrm{log}\,{p}_{x+y}(k)$	1
Sum of squares	$\quad \sum _{i\mathrm{=1}}^{N}\sum _{j\mathrm{=1}}^{N}{(i-\mu )}^{2}p(i,j)$	1
Sum variance	$\quad \sum _{k\mathrm{=2}}^{2N}{(k-{\mu }_{x+y})}^{2}{p}_{x+y}(k)$	1

**Figure 5:**
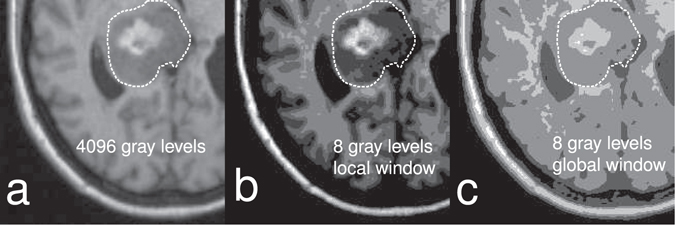
The effect of using different minimum and maximum values when quantizing the image. The images show how different minimum and maximum values influence the result when quantizing the original image, prior to constructing the GLCM. (**a**) Shows the original image with 4096 gray levels. In (**b**) the image has been quantized to 8 gray levels, and the minimum and maximum gray levels have been set to that of the ROI, dashed outline. In (**c**), the image has been quantized to 8 gray levels and minimum and maximum gray levels have been set based on the entire image. There are large regions of uniform gray levels in (**c**), the texture is very different compared to (**b**), and the only difference is how the maximum and minimum gray levels were chosen.

Medical images usually contain 1000 s of gray levels, which would result in a very large and sparse GLCM. The images need to be quantized to a lower number of gray levels, usually in the range 8–128 to obtain GLCMs that are densely populated and still capture the textures in the image. The minimum and maximum gray levels are also important when an image is quantized. They can be set in different ways, depending on if the image is scaled globally (i.e. using the global maximum and minimum) or locally (e.g. using the minimum and maximum gray level of the ROI, or set arbitrarily to enhance a specific feature in the image). This can result in very different textures, as shown in Fig. 5.

## Methods

### Patients

The image data used in this study was collected within two clinical trials, both with the aim to investigate use of image-based biomarkers. The clinical trials were approved by the Regional Ethical Review Board of Umeå University, and oral and written consent was given by all subjects.

#### Glioma patients

Eighteen patients (17 men, mean age 61, age range 42–75 and 1 woman, age 48) with high-grade inoperable glioma who received conformal radiotherapy with 2 Gy fractions for a total of 60 Gy concomitant with temozolomide^30^ were included in the study. The cohort comprised 26 lesions, imaged 2–4 times over the course of six weeks of radiotherapy for a total of 72 included exams.

#### Prostate patients

Eleven patients (mean age 67, age range 59–75) diagnosed with high risk prostate cancer according to the D’Amico criteria^31^ were included in the study. The patients received dose escalated radiotherapy of 78 Gy to the prostate with 50 Gy to the seminal vesicles and the pelvic lymph nodes. The cohort was imaged 1–3 times during the seven weeks of radiotherapy, and with followup exams at six months after radiotherapy. In total, 36 exams were used in this study.

### Imaging

All images were acquired on a Siemens Espree 1.5 T scanner (Siemens, Erlangen, Germany) using a 12 channel head coil for the glioma exams and a 6 channel body matrix array in combination with the spine coil for the prostate exams. Diffusion weighted images (DWI) were collected with a twice refocused spin echo sequence^32^ with echo-planar readout.

#### Glioma data set

Six b-values (0, 200, 400, 600, 800, 1000 s/mm^2^) were acquired with TR of 4000 ms, TE of 114 ms using four signal averages and a receiver bandwidth of 840 Hz/pixel. The acquired matrix size was 192 × 192, the voxel size was 1.2 × 1.2 mm^2^ and 19 slices were acquired with a slice thickness of 3.0 mm with a 0.9 mm slice gap. Tumors were delineated by a radiation oncologist on T1 weighted contrast-enhanced magnetization-prepared rapid gradient-echo (MPRAGE) images. Each tumor volume, consisting of one or more slices, was considered an ROI.

#### Prostate cancer data set

Two b-values, (0, 800 s/mm^2^) were acquired, with TR of 4000 ms, TE of 87 ms using 10 signal averages and a receiver bandwidth of 1116 Hz/pixel. The acquired matrix size was 160 × 136, the voxel size was 1.625 × 1.625 mm^2^, 20 slices were acquired with a slice thickness of 3.6 mm with no slice gap. The gross tumor volume (GTV) delineation for each patient was used as the ROI in the texture analysis.

### Data analysis

We explored four imaging and pre-processing parameters for the prostate cancer data set: image resolution, noise, the number of quantization gray levels and quantization method. For the glioma data set we also varied the choice of b-values used to construct the ADC map, for a total of five parameters.

**Figure 6:**
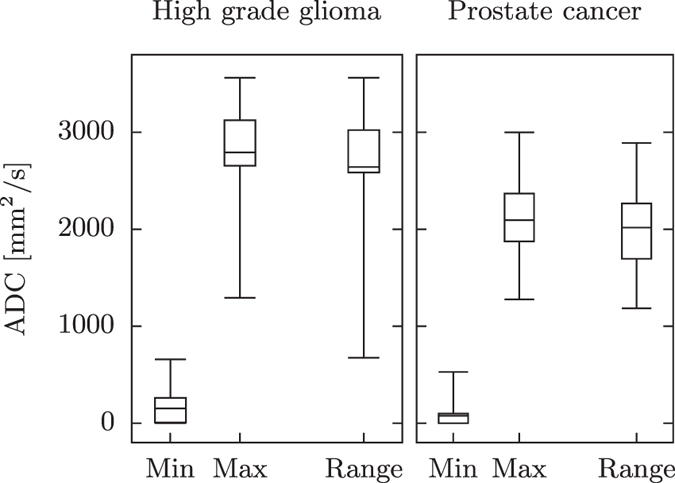
The span of ADC values in the data sets. Boxplots of ADC minimum and maximum values as well as the range of ADC values within each tumor for the glioma data set and the prostate cancer data set respectively.

To explore how the choices of imaging and pre-processing parameters affect the textures features we applied the same analysis work-flow while changing each of the parameters according to Table 4. Each row represent one investigated parameter, and the five pre-processing steps performed prior to calculating the texture features are shown in the columns. Only one parameter was changed at a time, while the others were held fixed at the reference settings. The resolution was changed by resampling the DWI prior to calculating the ADC such that the native pixel size was increased by a factor of 1.5 and 3.0. The noise standard deviation present in the images were *σ* = 17 for the glioma data and *σ* = 2.5 for the prostate cancer data. Gaussian noise with zero mean, generated using the Mersenne Twister random number generator, was added prior to calculating the ADC so that the noise in the images were increased by a factor of 2.0 and 4.0. The SNR of the images was sufficiently high that the assumption of a Gaussian noise distribution was valid^33^. Three quantization methods were tested. AutoROI sets the upper and lower intensity limits to the minimum and maximum values of the ROI. AutoSlice sets the limits to the minimum and maximum values of each slice of the ROI, prior to calculating the GLCM for that slice. Finally we used a manual method, were the minimum and maximum values were set to 0 and 3000 mm^2^/s for gliomas and 0 and 2400 mm^2^/s for prostate cancers, respectively. This represents the lower quartile of the minimum values in the ROIs, and the upper quartile of the maximum values for each data set, see Fig. 6. Four different combinations of b-values were used to create the ADC maps in the glioma data set: 0 and 1000 s/mm^2^, 200 and 1000 s/mm^2^, 0 to 1000 s/mm^2^ in steps of 200 s/mm^2^ and 200 to 1000 s/mm^2^ in steps of 200 s/mm^2^. The ADC map was calculated using a linear regression of the logarithm of the signal to the b-values.

**Table 4:** The pre-processing work flow and settings for each investigated parameter for the glioma data.

Investigated parameter	Step 1: Resolution of DWI	Step 2: Noise in DWI	Step 3: Calculate ADC	Step 4: Select GLCM size	Step 5: Quantize image
Resolution	1.2 mm^2^ (1.0×)	*σ* = 17	200–1000 s/mm^2^	32	AutoROI
	1.8 mm^2^ (1.5×)				
	3.6 mm^2^ (3.0×)				
Noise	1.2 mm^2^	*σ* = 17 (1.0×)	200–1000 s/mm^2^	32	AutoROI
		*σ* = 34 (2.0×)			
		*σ* = 68 (4.0×)			
Diffusion b-values	1.2 mm^2^	*σ* = 17	200–1000 s/mm^2^	32	AutoROI
			0–1000 s/mm^2^		
			200,1000 s/mm^2^		
			200,1000 s/mm^2^		
Gray levels	1.2 mm^2^	*σ* = 17	200–1000 s/mm^2^	8	AutoROI
				16	
				32	
				64	
				128	
Quantization method	1.2 mm^2^	*σ* = 17	200–1000 s/mm^2^	32	AutoROI
					AutoSlice
					Manual

Each row represents the work flow of one investigated parameter. The prostate cancer data set used a similar work flow, where the native resolution was 1.625 mm^2^, the native noise standard deviation was *σ* = 2.5, and the ADC was calculated using 0, 800 s/mm^2^ only.

Symmetric GLCMs were created for each slice by considering the closest neighbor in all eight directions (left, right, up, down and four diagonal directions). All GLCMs from one ROI were added before normalization, resulting in a directionally independent GLCM for each ROI.

We used two-sample Kolmogorov-Smirnov tests^34, 35^ to investigate if the texture feature distributions were significantly different when changing resolution, noise, diffusion b-values, number of gray levels and quantization method. Every combination of parameter setting pairs were compared. Resolution, noise and quantization method have 3 unique pairs of settings, combination of b-values have 6 pairs for glioma, and gray levels have 10 unique pairs of settings. For 19 texture features there were 19 × (3 + 3 + 3 + 6 + 10) = 475 test for the glioma data set and 19 × (3 + 3 + 3 + 10) = 361 test for the prostate data set, for a total of 836 test. Each test was performed at *α* = 0.01 and Bonferroni corrected for 836 tests. If any pair of settings resulted in a significant difference in a texture feature, the parameter was deemed to significantly affect that texture feature.
